# Eicosapentaenoic Acid Attenuates Inflammation in an LPS-Induced Mouse Model of Mastitis Partly Through Modulation of the PPARγ–NF-κB Signaling Pathway

**DOI:** 10.3390/biom16040592

**Published:** 2026-04-16

**Authors:** Zhiwei Duan, Ting Lu, Kejiang Liu, Xiaoxuan Zhao, Wenkai Bai, Bohao Zhang, Quanwei Zhang, Xingxu Zhao, Weitao Dong, Yong Zhang

**Affiliations:** 1 College of Veterinary Medicine, Gansu Agriculture University, Lanzhou 730070, China; 2 Gansu Key Laboratory of Animal Generational Physiology and Reproductive Regulation, Lanzhou 730070, China; 3 College of Life Sciences and Biotechnology, Gansu Agricultural University, Lanzhou 730070, China

**Keywords:** mastitis, Eicosapentaenoic acid, PPARγ, NF-κB

## Abstract

Mastitis is a common inflammatory disease that harms mammary gland health. Its development is closely linked to dysregulated inflammatory signaling. Eicosapentaenoic acid (EPA), an omega-3 polyunsaturated fatty acid, has potential anti-inflammatory effects. However, its molecular mechanism in mastitis prevention remains unclear. In this study, we used both in vivo and in vitro models to evaluate how EPA pretreatment regulates mastitis-related inflammatory signaling. Transcriptome analysis showed that differentially expressed genes after EPA treatment were mainly enriched in the peroxisome proliferator-activated receptor (PPAR) signaling pathway. In an LPS-induced mastitis model, EPA restored the LPS-reduced PPARγ protein level and suppressed NF-κB p65 activation, consistent with reduced nuclear translocation of p65. Similar effects were observed in mammary epithelial cells, where EPA inhibited NF-κB activation at 50 and 100 μM. Functional experiments further showed that a PPARγ agonist mimicked the inhibitory effect of EPA on p65, whereas PPARγ antagonist partially abrogated EPA-mediated inhibition of p65. Collectively, these data indicate that EPA attenuates mastitis-associated inflammation at least in part through the PPARγ–NF-κB axis.

## 1. Introduction

Mastitis is a common inflammatory disease during lactation, with a relatively high incidence rate of 2–4% [1]. Typical symptoms include breast swelling, pain, and fever, and severe cases may involve systemic inflammation [2]. Mastitis can disrupt breastfeeding and reduce quality of life. It also tends to recur, and repeated inflammation can cause lasting damage to mammary tissue. Several epidemiological and clinical studies have reported an association between prior mastitis and subsequent breast cancer risk [3,4,5,6]. Therefore, safe interventions that can be used long term are needed to protect mammary gland health during lactation. In recent years, nutritional intervention has gained attention because it is generally safe and suitable for long-term use. Eicosapentaenoic acid (EPA), a common omega-3 polyunsaturated fatty acid, has been reported to regulate inflammation and immune function, suggesting a possible strategy for mastitis intervention [7].

Sustained inflammatory signaling is a key feature of mastitis. The NF-κB pathway is one of the central pathways that regulate inflammation [8]. After activation, NF-κB drives the expression of pro-inflammatory cytokines such as TNF-α, IL-1β, and IL-6, and it also induces inflammatory enzymes such as COX-2 and iNOS. This amplifies local inflammation and aggravates tissue injury [9]. Therefore, early inhibition of NF-κB-driven pro-inflammatory transcription is considered an important strategy to prevent excessive inflammation. Current clinical treatment of mastitis mainly relies on antibiotics. However, antibiotics primarily target pathogens and have limited direct effects on host inflammatory signaling. Repeated or long-term use may also increase antibiotic resistance and may pose risks to maternal and infant health [10,11]. Thus, alternative strategies that modulate host immune responses and complement conventional antibiotic therapy are increasingly recognized as important approaches to address antimicrobial resistance [12].

Peroxisome proliferator-activated receptors (PPARs) are nuclear receptors that are closely related to lipid metabolism and inflammatory regulation [13,14]. PPARγ is a major isoform. It can be activated by endogenous fatty acids and their metabolites [15]. It plays key roles in metabolic homeostasis and inflammation control [16]. Previous studies show that PPARγ activation can negatively regulate NF-κB-mediated pro-inflammatory gene expression. This can occur through inhibition of p65 nuclear translocation, reduction of NF-κB transcriptional activity, or competition for co-activators [17]. Many studies have also suggested that the PPARγ/NF-κB axis is important in mastitis-related inflammation [18,19,20,21]. However, most work has focused on drugs or molecular interventions. It is still unclear whether a safer, long-term nutritional approach can regulate this pathway in an upstream manner.

EPA has been reported to act as a ligand of PPARγ and may modulate PPAR signaling through nutritional intervention [22]. Studies report that EPA supplementation during pregnancy and lactation can improve maternal inflammatory status. Changes in breast milk composition may also benefit immune development and inflammatory responses in offspring [23,24]. These findings suggest that maternal nutrition may influence local inflammation in the mammary gland. Some studies using DHA or other omega-3 fatty acids have observed changes in PPARγ/NF-κB-related markers in mastitis models [25,26]. However, these studies were often limited to in vitro systems or a small set of molecular endpoints. For EPA-based prevention strategies, systematic pathway-level evidence and functional validation of the PPARγ–NF-κB axis are still limited. Therefore, we selected EPA as a representative omega-3 PUFA and evaluated its regulatory effects on key inflammatory signaling before mastitis onset.

In this study, we focused on EPA pretreatment and used both in vivo and in vitro models. We assessed whether nutritional intervention can regulate key inflammatory signaling pathways before mastitis develops. We used transcriptome analysis to evaluate global signaling changes after EPA pretreatment. We then performed molecular and functional experiments to test whether PPARγ is involved in EPA regulation of p65-driven inflammatory responses. Our findings may provide experimental support for nutritional prevention strategies against mastitis.

## 2. Materials and Methods

### 2.1. Experimental Design

All animal procedures were approved by the Animal Care and Use Committee of Gansu Agricultural University (approval number: GSAC-Eth-VMC-2024-031, 16 January 2024). SPF female Kunming mice (8 weeks old, 35 ± 2 g) were purchased from the Laboratory Animal Center of the Lanzhou Veterinary Research Institute, Chinese Academy of Agricultural Sciences, Lanzhou, China. Mice were housed under SPF conditions (21 ± 2 °C, 50 ± 5% humidity) with a 12 h light/dark cycle, and had free access to sterilized feed and water. Mice were acclimated for one week and trained for oral gavage. After acclimation, males and females were housed together for mating at a ratio of 1:3. EPA (70% purity) was purchased from Shaanxi Benhe Biotechnology Co., Ltd., Xi’an, China.

Pregnant mice were randomly assigned to five groups (*n* = 12 per group): (1) Control; (2) LPS; (3) Vehicle; (4) EPA low dose (EPA-L); (5) EPA high dose (EPA-H). All reported *n* values represent independent biological replicates. Differences in sample size among assays reflect differences in tissue availability and assay requirements. Histological scoring and Western blot analyses were performed on the same subset of mammary tissue samples with matched tissue sections and protein extracts, whereas RT-qPCR could be performed on a slightly larger number of samples because RNA extraction required less starting material. From the gavage training period to one day before euthanasia, mice received daily oral gavage of EPA at 150 mg/kg (EPA-L) or 300 mg/kg (EPA-H). The Vehicle group received the same volume of soybean oil (Solarbio, Beijing, China). The other groups received the same volume of purified water.

Mastitis was induced on postpartum day 7. The Control group received an intraductal injection of 50 μL sterile PBS (Solarbio, Beijing, China) into the fourth pair of mammary glands. All other groups received the same volume of 0.2 mg/mL LPS (Solarbio, Beijing, China) [27], a dose selected based on a previously validated mouse model of LPS-induced mastitis. After injection, the mammary gland was gently massaged to distribute the solution. Mice were euthanized 24 h after LPS injection by CO_2_ inhalation. Mammary tissues were collected. One portion was snap-frozen in liquid nitrogen and stored at −80 °C for molecular assays. The remaining tissue was fixed in 4% paraformaldehyde (Solarbio, Beijing, China) for histology and immunostaining.

### 2.2. Cell Culture and Treatment

The mouse mammary epithelial cell line HC-11 (CL-0616) was obtained from Procell (Wuhan, China). HC-11 cells were used as a murine mammary epithelial model because they retain key epithelial characteristics and have been widely used to study inflammatory signaling in mammary cells. Cells were cultured in RPMI 1640 (Gibco, Grand Island, NY, USA) supplemented with 10% fetal bovine serum (Invigentech, Houston, TX, USA) and 1% penicillin–streptomycin (Gibco, Grand Island, NY, USA) at 37 °C with 5% CO_2_.

To determine the LPS concentration for establishing the inflammatory model, HC-11 cells were first exposed to different concentrations of LPS (1, 10, 20, 50 and 100 μg/mL). Cell viability was assessed using a CCK-8 assay. No significant change in viability was observed at 1–20 μg/mL, whereas a significant reduction was first detected at 50 μg/mL. Therefore, 50 μg/mL LPS was selected for subsequent experiments under the present conditions.

To assess the effects of EPA on HC-11, cells were pretreated with EPA (25, 50, 100 and 200 μM) for 12 h, followed by stimulation with LPS (50 μg/mL; Solarbio, Beijing, China) for 24 h to establish an in vitro inflammatory model. The LPS dose was selected based on a preliminary dose–response test. To examine the role of PPARγ, a selective agonist, pioglitazone (10 μM; #S2590, Selleck, Houston, TX, USA), and an antagonist, GW9662 (10 μM; #S2915, Selleck, Houston, TX, USA), were used. These concentrations were selected based on previously reported conditions in mastitis-related or epithelial cell models investigating PPARγ signaling [18,28]. Pioglitazone was applied during the pretreatment period to phenocopy the effect of EPA, followed by LPS stimulation. GW9662 was applied 1 h before EPA pretreatment, followed by EPA pretreatment for 12 h and then LPS stimulation, to test whether PPARγ inhibition reduces the protective effect of EPA. All compounds were dissolved in DMSO (Solarbio, Beijing, China), and the final DMSO concentration was kept below 0.1%.

### 2.3. Cell Viability Assay

HC-11 cells were seeded in 96-well plates at 5 × 10^3^ cells per well. After reaching 70% confluence, cells were treated as designed. CCK-8 reagent (P-CA-001, Procell, Wuhan, China) was added and incubated for 1 h at 37 °C in the dark. Absorbance was measured at 450 nm using a microplate reader (Model 680, Bio-Rad Laboratories, Hercules, CA, USA).

### 2.4. Transcriptome Sequencing Analysis

Mammary tissues from the control, LPS, and high-dose EPA groups were subjected to RNA sequencing. Three biological replicates per group were used for RNA sequencing. RNA sequencing of mammary tissue was performed by Shanghai Personal Biotechnology Co., Ltd., Shanghai, China. Total RNA was extracted, and mRNA was enriched using Oligo(dT) magnetic beads. mRNA was fragmented to 300 bp and reverse-transcribed into cDNA. Libraries were constructed and sequenced on an Illumina NovaSeq 6000 platform (Illumina, San Diego, CA, USA). Raw data were quality-controlled before downstream analysis. Bioinformatics analysis was performed on the Personal Gene Cloud platform (https://www.genescloud.cn, accessed on 10 January 2026). Differentially expressed genes were defined using the criteria |Log_2_ FoldChange| > 1 and adjusted *p* < 0.05.

### 2.5. Histological Analysis

Paraffin-embedded mammary tissues fixed in 4% paraformaldehyde were sectioned at 4 μm and stained with a hematoxylin and eosin (H&E) staining kit (Solarbio, Beijing, China). Images were captured using a Zeiss microscope (Axiocam 208 color, Zeiss, Oberkochen, Germany). Image analysis was performed using ImageJ software (version 1.53, National Institutes of Health, Bethesda, MD, USA). Pathological scoring was performed according to a previously reported method with minor modifications, based on the degree of inflammatory cell infiltration, alveolar wall thickening, interstitial edema, and structural damage [29]. Each parameter was graded on a 0–5 scale (0, no damage; 1, mild damage; 2, moderate damage; 3, severe damage; 4, very severe damage; and 5, necrosis), and the total score was used for statistical analysis. Scoring was performed by two observers in a blinded manner.

### 2.6. Real-Time qPCR

Total RNA was extracted from mammary tissue using Trizol reagent (Solarbio, Beijing, China) and reverse-transcribed into cDNA using an Evo M-MLV RT kit (Abio, Shanghai, China). qPCR was performed on a LightCycler 96 system (Roche, Basel, Switzerland) using a 2× SYBR Green Pro Taq HS premix (Selleck, Houston, TX, USA). β-actin was used as the reference gene. Relative expression was calculated using the 2^−ΔΔCt^ method. Primer sequences are listed in Supplementary Table S1.

### 2.7. Western Blot Analysis

Total protein from tissues and cells was extracted using ice-cold RIPA lysis buffer (Solarbio, Beijing, China). Proteins were separated by SDS-PAGE and transferred onto PVDF membranes (Millipore, Billerica, MA, USA). Membranes were blocked with 5% non-fat milk (Solarbio, Beijing, China) and incubated with primary antibodies at 4 °C overnight, followed by HRP-conjugated secondary antibodies for 1 h at room temperature (Affinity Biosciences, Changzhou, China; S0001, 1:5000). Membranes were washed with PBST between steps. Bands were detected using an ECL kit (#G2020, Servicebio, Wuhan, China) and imaged using a chemiluminescence system (SCG-W3000, Servicebio, Wuhan, China). β-actin was used as the loading control. Band intensity was quantified using ImageJ software (version 1.53, National Institutes of Health, Bethesda, MD, USA). Antibody information is provided in Supplementary Table S2.

### 2.8. Immunofluorescence Staining

Immunofluorescence was used to detect the expression and localization of PPARγ and p65 in mammary tissue sections and HC-11 cell coverslips. DAPI (Solarbio, Beijing, China) was used for nuclear staining. Samples were mounted with an anti-fade mounting medium (Solarbio, Beijing, China) and imaged with an inverted fluorescence microscope (Revolve Omega, Apexbio, Suzhou, China).

### 2.9. Statistical Analysis

All analyses were performed using GraphPad Prism 10.1.2 (GraphPad Software, La Jolla, CA, USA). Data are presented as mean ± SEM. Comparisons among multiple groups were performed using one-way ANOVA followed by Tukey’s post hoc test. A *p* value < 0.05 was considered statistically significant.

## 3. Results

### 3.1. EPA Alleviates LPS-Induced Mammary Tissue Injury and Inflammatory Responses In Vivo

**Figure 1:**
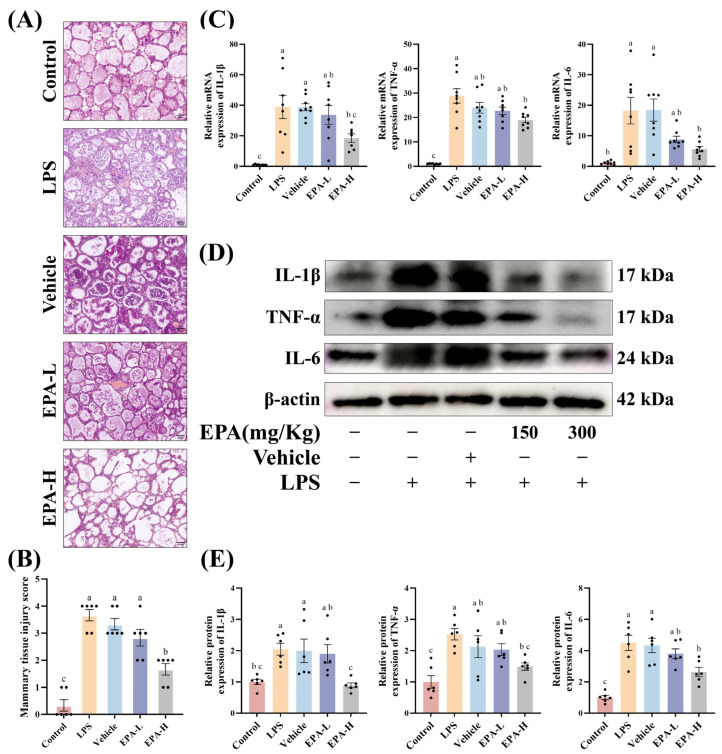
EPA alleviates LPS-induced mammary tissue injury and inflammatory responses in vivo. (**A**) Representative images of mammary gland sections stained with H&E. Scale bars: 50 μm. (**B**) Mammary tissue injury score (*n* = 6, assessed in a blinded manner.) (**C**) mRNA expression of TNF-α, IL-1β, and IL-6 in mammary tissues measured by RT-qPCR (*n* = 8). (**D**,**E**) Western blot analysis of TNF-α, IL-1β, and IL-6 protein expression in mammary tissues (*n* = 6). Different superscripts indicate significant differences (*p* < 0.05). (Original Western blots can be found in Supplementary Figure S1).

To evaluate the effects of EPA on mammary tissue injury and inflammation, we performed histological and molecular analyses. H&E staining showed that, compared with the Control group, the LPS group had thickened alveolar walls and marked inflammatory cell infiltration. After EPA pretreatment, these pathological changes were reduced, inflammatory cell infiltration decreased, and tissue structure was more preserved. The pathological score also significantly decreased (Figure 1A,B). We then measured inflammatory cytokines in mammary tissue. qPCR showed that TNF-α, IL-1β, and IL-6 mRNA levels were significantly increased in the LPS group, while EPA pretreatment markedly reduced these cytokines (Figure 1C). Western blot results were consistent and showed reduced TNF-α, IL-1β, and IL-6 protein levels after EPA pretreatment (Figure 1D,E). These results suggest that EPA reduces LPS-induced mammary tissue injury and suppresses local inflammation.

### 3.2. Transcriptomic Analysis Identifies Inflammation-Related Pathways Regulated by EPA in LPS-Induced Mastitis

**Figure 2:**
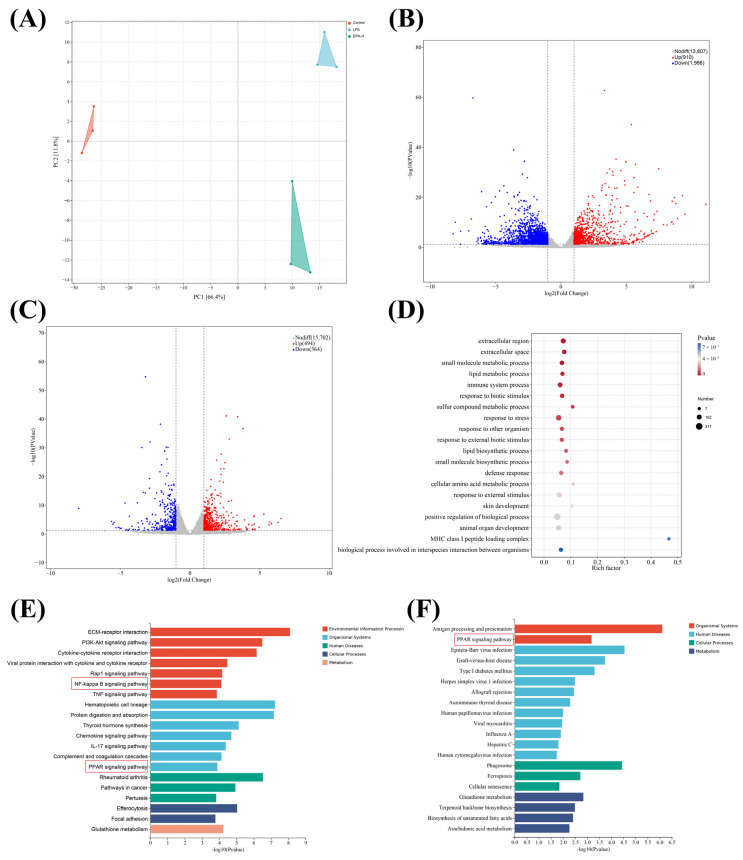
Transcriptomic analysis identifies inflammation-related pathways regulated by EPA in LPS-induced mastitis. (**A**) PCA shows distinct separation among Control, LPS, and EPA-H groups. (**B**) Volcano plot shows 2896 DEGs between LPS and Control. (**C**) Volcano plot shows 858 DEGs between EPA-H and LPS. (**D**) GO enrichment analysis of DEGs between LPS and EPA-H groups. (**E**) KEGG pathway analysis of DEGs between LPS and Control. (**F**) KEGG pathway analysis of DEGs between EPA-H and LPS.

PCA showed clear separation among the Control, LPS, and EPA-H groups (Figure 2A), indicating distinct gene expression patterns. Differential expression analysis supported this finding. Compared with the Control group, 2896 DEGs were identified in the LPS group, including 910 upregulated and 1986 downregulated genes (Figure 2B). Compared with the LPS group, 858 DEGs were identified in the EPA-H group, including 494 upregulated and 364 downregulated genes (Figure 2C). GO analysis of DEGs between the LPS and EPA-H groups showed enrichment in immune system processes, stress responses, and lipid metabolism-related processes (Figure 2D). This suggests that EPA may regulate inflammation through immune and metabolic functions. KEGG analysis indicated that LPS-induced DEGs were enriched in multiple inflammation-related pathways, including PPAR and NF-κB signaling (Figure 2E). After EPA treatment, DEGs were significantly enriched in the PPAR pathway compared with the LPS group (Figure 2F). These results suggest that PPAR-related signaling may contribute to the response to EPA during mastitis.

### 3.3. EPA Suppresses NF-κB Activation and Restores PPARγ Expression in Mammary Tissue

**Figure 3:**
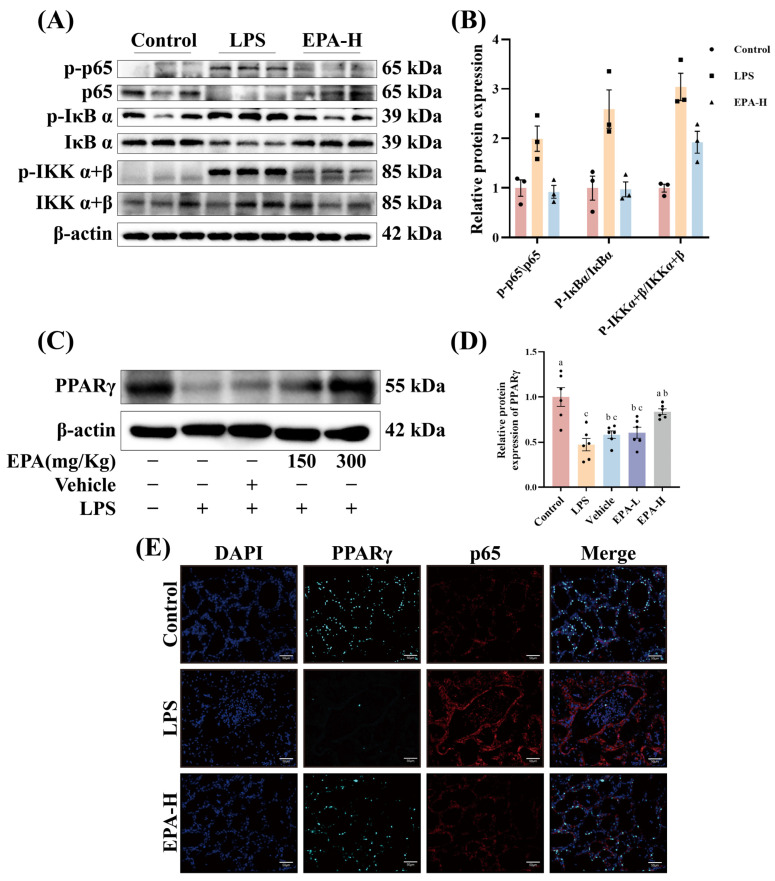
EPA Suppresses NF-κB Activation and Restores PPARγ Expression in Mammary Tissue. (**A**,**B**) Western blot analysis of p65, IκB α, and IKK α + β protein expression in mammary tissues (*n* = 3). (**C**,**D**) Western blot analysis of PPARγ protein expression in mammary tissues (Original Western blots can be found in Supplementary Figure S1). (*n* = 6). (**E**) Immunofluorescence analysis of p65 and PPARγ expression in mammary tissues: nuclei (blue), PPARγ (cyan), p65 (red), and merged PPARγ and p65 staining. Scale bars: 50 μm. Different superscripts indicate significant differences (*p* < 0.05).

In the in vivo experiment, NF-κB-related analyses were further conducted in the high-dose EPA group, because this dose showed the most evident protective effects on tissue injury and inflammatory markers. Compared with the Control group, NF-κB activation was increased in mammary tissue of the LPS group. This was evidenced by increased p65 phosphorylation along with altered IκBα and IKKα/β phosphorylation. EPA pretreatment significantly reduced NF-κB activation (Figure 3A,B). At the same time, EPA reversed the LPS-induced decrease in PPARγ protein levels (Figure 3C,D). Immunofluorescence showed stronger p65 signals and lower PPARγ signals in the LPS group. In contrast, high-dose EPA reduced NF-κB signals and restored PPARγ expression (Figure 3E). These results suggest that EPA suppresses NF-κB signaling in mammary tissue and is associated with restoration of PPARγ expression.

### 3.4. EPA Attenuates LPS-Induced Inflammatory Responses in HC-11 Mammary Epithelial Cells

**Figure 4:**
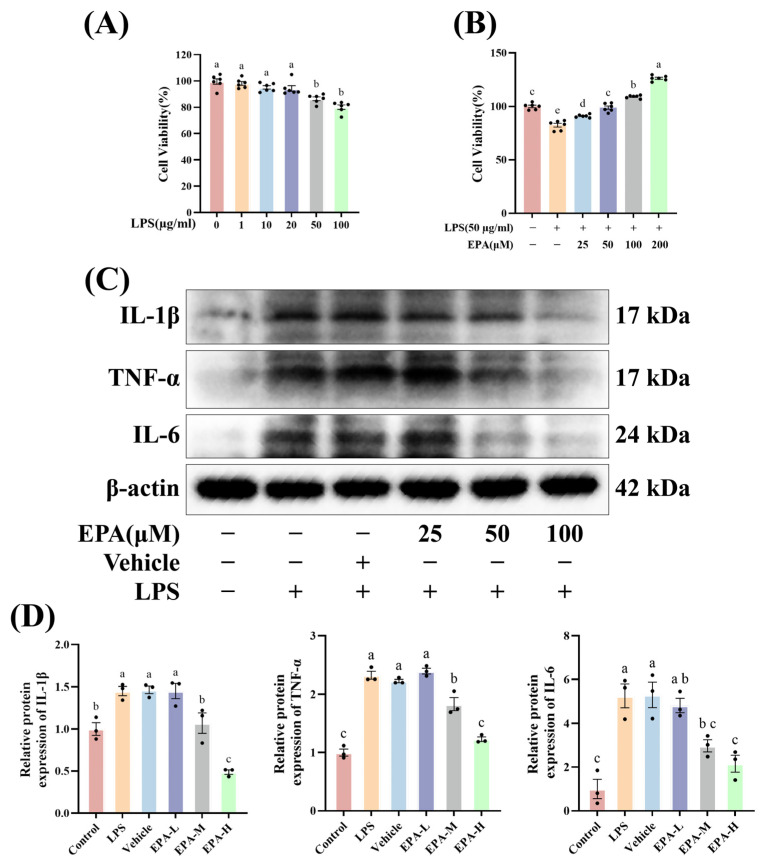
EPA attenuates LPS-induced inflammatory responses in HC-11 mammary epithelial cells. (**A**) CCK-8 assay assessing the effects of different concentrations of LPS on HC-11 cell viability (*n* = 6). (**B**) CCK-8 assay evaluating the effects of increasing concentrations of EPA on HC-11 cell viability in the presence of LPS (*n* = 6). (**C**,**D**) Western blot analysis of inflammatory cytokine protein expression in HC-11 cells following LPS stimulation and EPA treatment (*n* = 3). Different superscripts indicate significant differences (*p* < 0.05). (Original Western blots can be found in Supplementary Figure S1).

To establish an in vitro model, we first tested the effects of different LPS concentrations on HC-11 cell viability. Cell viability decreased with increasing LPS concentration. LPS at 50 μg/mL caused a significant reduction, so this dose was used for later experiments (Figure 4A). We then tested the effects of EPA (25, 50, 100, and 200 μM). CCK-8 results showed no obvious cytotoxicity within this range, and EPA partially improved LPS-induced viability reduction (Figure 4B). Western blot showed that LPS increased TNF-α, IL-1β, and IL-6 protein levels, while EPA significantly reduced these cytokines. A clear effect was observed at 50 μM EPA (Figure 4C,D). These results indicate that EPA reduces LPS-induced inflammatory responses in vitro.

### 3.5. EPA Suppresses NF-κB Activation, Accompanied by Restoration of PPARγ Expression

**Figure 5:**
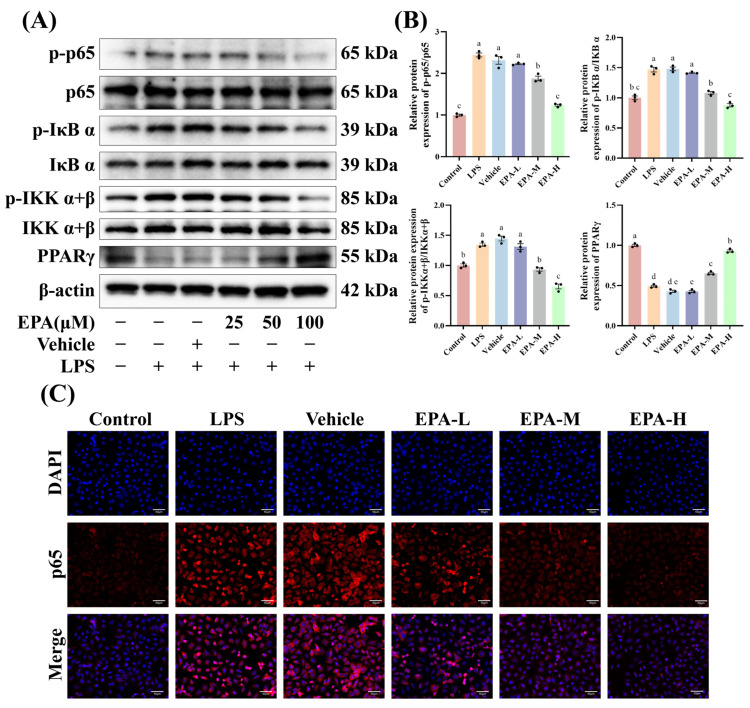
EPA suppresses NF-κB activation, accompanied by restoration of PPARγ expression. (**A**,**B**) Western blot analysis of NF-κB pathway related proteins and PPARγ expression in HC-11 cells following LPS stimulation and EPA treatment (*n* = 3). (Original Western blots can be found in Supplementary Figure S1). (**C**) Immunofluorescence analysis of p65 localization in HC-11 cells following LPS and EPA treatment: nuclei (blue), p65 (red), and merged DAPI and p65 staining. Scale bars: 50 μm. Different superscripts indicate significant differences (*p* < 0.05).

Western blot results showed that LPS strongly activated the NF-κB pathway in HC-11 cells, with increased phosphorylation of p65, IκBα, and IKKα/β. EPA inhibited LPS-induced NF-κB activation at 50 and 100 μM, whereas no significant effect was observed at 25 μM. EPA also restored PPARγ protein levels (Figure 5A,B). Immunofluorescence further showed that LPS promoted p65 nuclear translocation, while EPA reduced p65 signals and inhibited its nuclear localization (Figure 5C). These results suggest that EPA suppresses NF-κB activation in vitro and is associated with increased PPARγ expression.

### 3.6. Functional Involvement of PPARγ in EPA-Regulated NF-κB Signaling

**Figure 6:**
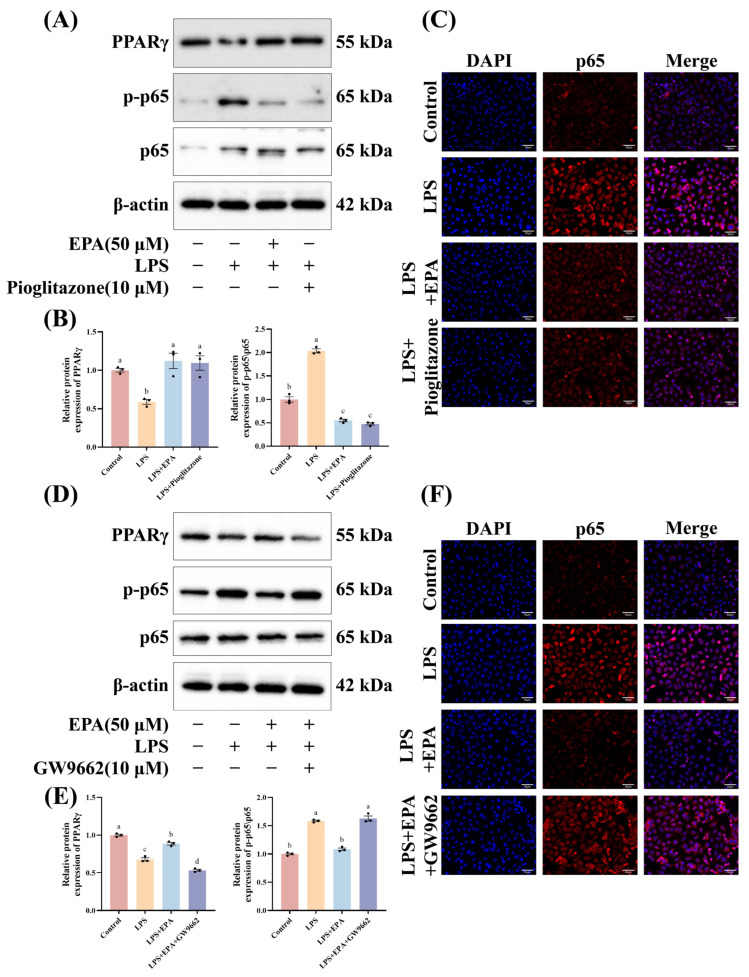
Functional involvement of PPARγ in EPA-regulated NF-κB signaling. (**A**,**B**) Western blot analysis of PPARγ and p65 following treatment with PPARγ agonist (*n* = 3). (**C**) Immunofluorescence analysis of p65 localization after PPARγ activation: nuclei (blue), p65 (red), and merged DAPI and p65 staining. Scale bars: 50 μm. (**D**,**E**) Western blot analysis of PPARγ and p65 following PPARγ antagonist treatment in the presence of EPA (*n* = 3). (Original Western blots can be found in Supplementary Figure S1). (**F**) Immunofluorescence analysis of p65 localization under PPARγ inhibition and EPA treatment: nuclei (blue), p65 (red), and merged DAPI and p65 staining. Scale bars: 50 μm. Different superscripts indicate significant differences (*p* < 0.05).

To further verify whether PPARγ functionally mediates the inhibitory effect of EPA on NF-κB signaling, we performed functional experiments using a PPARγ agonist and antagonist in HC-11 cells. In the agonist experiment, Western blot showed that the agonist increased PPARγ protein levels and reduced the p-p65/p65 ratio (Figure 6A,B). Immunofluorescence also showed reduced nuclear localization of p65 (Figure 6C), suggesting that activation of PPARγ mimics the inhibitory effect of EPA on NF-κB signaling. In the antagonist experiment, inhibition of PPARγ reduced PPARγ protein levels and weakened the inhibitory effect of EPA on p65, with increased p65 phosphorylation (Figure 6D,E). Consistently, immunofluorescence analysis showed enhanced nuclear localization of p65 under PPARγ inhibition (Figure 6F). Taken together, these findings suggest that PPARγ activity at least partly mediates the suppressive effect of EPA on NF-κB signaling.

## 4. Discussion

Mastitis development and progression are often accompanied by sustained activation of inflammatory signaling. Mammary epithelial cells are considered a major source of pro-inflammatory mediators during mastitis. Previous studies in mammary and other epithelial cell models show that inflammatory stimuli can rapidly activate pro-inflammatory pathways and induce TNF-α, IL-1β, and IL-6, which aggravates tissue injury [30]. Fatty acid-based interventions have also been reported to regulate inflammation through NF-κB signaling in mammary epithelial cells [31]. In subclinical mastitis models, inflammation is also linked to oxidative stress, autophagy, and blood–milk barrier damage [32]. Based on this background, we observed preventive effects of EPA in both in vivo and in vitro systems. Although mastitis was induced in lactating mice on postpartum day 7, the model mainly reflects LPS-induced inflammatory injury in mammary tissue. Lactation-related functional outcomes were not evaluated in this study. The consistency of the anti-inflammatory effects observed across the in vivo and in vitro models is in agreement with prior evidence that omega-3 PUFAs have anti-inflammatory potential [33].

Transcriptome analysis showed that EPA markedly altered gene expression patterns under inflammatory conditions. These changes involved not only inflammatory genes but also stress response and lipid metabolism pathways. Previous studies suggest that inflammation in mammary epithelial cells is often accompanied by lipid metabolism disorders. Altered fatty acid synthesis may further affect the strength and duration of inflammatory signaling [34]. Metabolic pathways can also regulate inflammation. For example, in bovine mammary epithelial cells, SDC3 can increase PPARγ through the AMPK/SIRT1 pathway and suppress NF-κB-related inflammation, thereby affecting milk fat synthesis and cytokine expression [35]. In our study, the NF-κB pathway showed clear changes. NF-κB is a key transcription factor that controls inflammatory gene expression. Its activation is commonly reflected by p65 translocation into the nucleus, which initiates transcription of pro-inflammatory genes. Reducing NF-κB activation and limiting nuclear p65 accumulation can effectively decrease inflammatory cytokine expression [36]. We found that EPA reduced NF-κB activation, decreased p65 nuclear localization, and lowered cytokine expression, supporting a regulatory role of EPA on NF-κB signaling. Because IκBα-related proteins were assessed at a single time point, the difference between phosphorylated and total IκBα should be interpreted cautiously and may not reflect the full dynamics of IκBα turnover. However, as the present study mainly evaluated changes in NF-κB signaling markers, further experiments directly manipulating NF-κB activity (e.g., p65 inhibition or knockdown) would be valuable to determine whether NF-κB signaling is functionally required for the inflammatory responses observed in this model.

PPARγ provided an important clue for understanding the anti-inflammatory effect of EPA. PPARγ is a nuclear receptor related to lipid metabolism and inflammation. Its activation in many disease models is associated with reduced inflammatory cytokines and inhibition of NF-κB activity [37,38,39]. In nervous system injury and inflammation, PPARγ activation is considered a key mechanism for the protective effects of several natural compounds [40]. In lactation-related studies, PPARγ plays a critical role in mammary inflammatory homeostasis and milk quality. In mice, mammary-specific deletion of maternal PPARγ increases inflammatory lipid production during lactation. This leads to inflammatory milk and induces systemic inflammation and abnormal development in nursing neonates [41].

Many studies show that PPARγ can suppress inflammation by regulating the NF-κB axis. In neuroinflammation and pain models, PPARγ-related interventions can inhibit NF-κB activation and reduce inflammatory responses [42]. In systemic inflammation, PPARγ agonists have been reported to improve tissue injury by regulating IκBα/p65 signaling [43]. Similar anti-inflammatory effects through PPARγ targeting have also been shown in other models [44]. In mastitis animal models, PPARγ agonists have been directly reported to prevent inflammatory responses, supporting the functional role of PPARγ in mastitis regulation [45]. In our study, EPA restored PPARγ expression. A PPARγ agonist reduced p65 activation and p65 nuclear localization even without EPA, while a PPARγ antagonist weakened these EPA effects. These functional data support that PPARγ contributes to EPA-mediated anti-inflammatory activity [46]. However, because the present evidence is based on pharmacological modulation and GW9662 only partially reversed the effect of EPA, these findings support the involvement of PPARγ rather than proving that EPA action is fully dependent on PPARγ. In addition, EPA may also influence inflammatory responses by altering the balance between ω-3 and ω-6 fatty acids and competing with arachidonic acid for eicosanoid synthesis, which could further contribute to the anti-inflammatory effects observed in this study.

It should be noted that EPA regulation of inflammation may not be limited to direct interaction between PPARγ and NF-κB. Other inflammatory pathways, such as MAPK/JNK/AKT signaling, may also contribute to the observed effects of EPA, but these pathways were not functionally examined in the present study. PPARγ is a key node connecting lipid metabolism and inflammation. Changes in its activity can reshape the local inflammatory environment by affecting lipid uptake, fatty acid metabolism, and energy homeostasis. For example, in NAFLD mice, mulberry extract improves lipid metabolism and reduces inflammation through the AMPK/PPARγ/NF-κB axis, suggesting that PPARγ effects on inflammation may partly depend on metabolic regulation [47]. In addition, EPA is a precursor of pro-resolving lipid mediators and may contribute to inflammation resolution. In experimental necrotizing enterocolitis, maternal omega-3 PUFA supplementation increases 18-HEPE in offspring tissues and reduces inflammation and tissue injury. Exogenous 18-HEPE can also be protective without maternal supplementation [48]. However, lipidomic analysis was not performed in the present study, and therefore the involvement of EPA-derived lipid mediators such as 18-HEPE remains speculative in our model. Although we did not directly test EPA metabolites, transcriptomic changes in lipid metabolism pathways suggest that EPA may act through both inflammatory regulation and metabolic remodeling.

In addition to mechanistic insights, the potential translational relevance of EPA should also be interpreted with caution. Although EPA and other omega-3 PUFAs show anti-inflammatory effects in many disease models, their clinical effects vary across diseases [49,50]. These differences may depend on disease type, metabolic and inflammatory status, formulation, dose, and study design. In addition, mastitis remains less well studied than many other inflammatory conditions in the context of omega-3 PUFA intervention. Therefore, this study should mainly be viewed as providing mechanistic evidence that EPA may act as a nutritional preventive strategy for mastitis, rather than direct proof of clinical efficacy. Further studies in clinically relevant mastitis models and human populations are needed to evaluate its translational value.

This study has several limitations. The LPS-based models mainly represent Gram-negative associated inflammation, and the relevance to Gram-positive mastitis requires further validation. In addition, PPARγ involvement was supported by pharmacological evidence but lacks direct genetic confirmation. Furthermore, inflammatory responses were mainly evaluated in mammary epithelial cells, whereas other immune cells, such as macrophages, also play important roles in mastitis-associated innate immunity. Nonetheless, the consistent findings obtained from both in vivo and in vitro experiments suggest that EPA may attenuate mastitis-associated inflammation, at least in part, through modulation of the PPARγ–NF-κB signaling axis. Therefore, the potential value of EPA may lie more in preventive nutritional support or adjunctive anti-inflammatory use alongside conventional antimicrobial therapy, rather than replacement of antibiotic treatment.

## 5. Conclusions

In summary, using transcriptome analysis, in vivo and in vitro phenotyping, and pharmacological experiments with a PPARγ agonist and antagonist, we found that EPA suppresses NF-κB signaling and p65 nuclear translocation, accompanied by restoration of PPARγ expression, thereby attenuating mastitis-associated inflammatory responses. Pharmacological evidence further supports that PPARγ is involved, at least in part, in the anti-inflammatory effects of EPA. These findings provide molecular support for the potential role of omega-3 PUFAs in mastitis prevention.

## Data Availability

The original contributions presented in this study are included in the article/Supplementary Material. Further inquiries can be directed to the corresponding authors.

## Supplementary Materials

The following supporting information can be downloaded at: https://www.mdpi.com/article/10.3390/biom16040592/s1, Figure S1: Whole Western blots; Table S1: The used antibodies and dilution ratio in the present study; Table S2: qRT-PCR primer sequences.
