# Supplementary material for: Eicosapentaenoic Acid Attenuates Inflammation in an LPS-Induced Mouse Model of Mastitis Partly Through Modulation of the PPARγ–NF-κB Signaling Pathway
Source: Biomolecules. 2026 Apr 16;16(4):592. doi: 10.3390/biom16040592 (PMC13115396; doi:10.3390/biom16040592)
Supplement: Supplementary file 1 [file biomolecules-16-00592-s001.zip › Figure S1 Whole Western blots.pdf]

Figure S1. Whole Western blots

1. Western blot data for mammary gland tissue. Lanes from left to right represent the control group, LPS group, Vehicle group, EPA-L group, and EPA-H group.

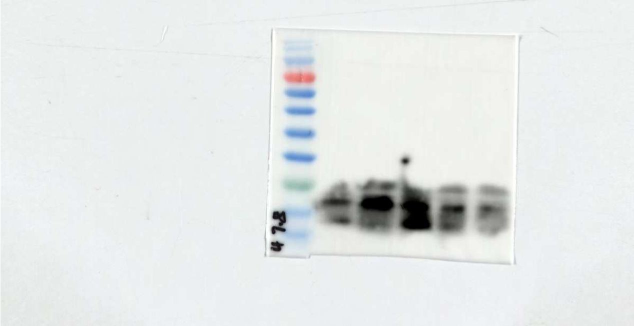

IL-1β

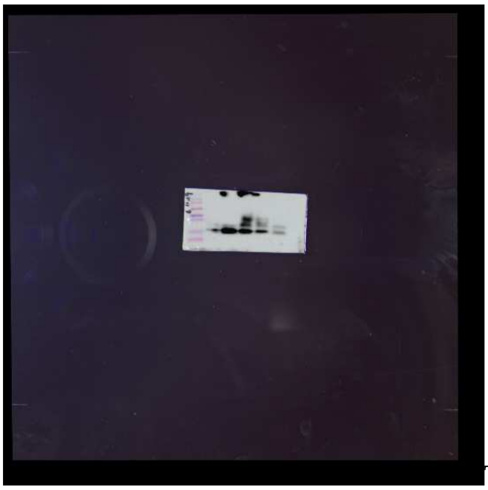

TNF-α

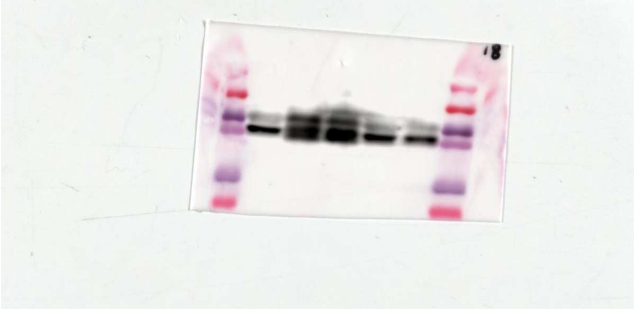

IL-6

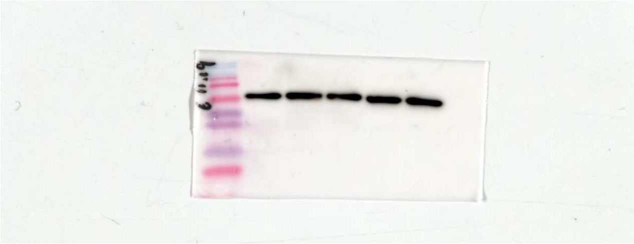

β-actin

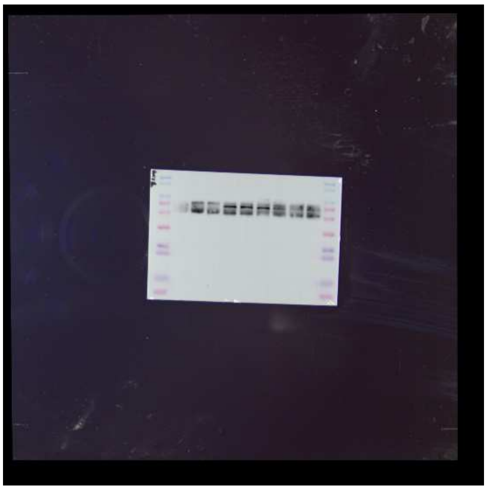
p-p65

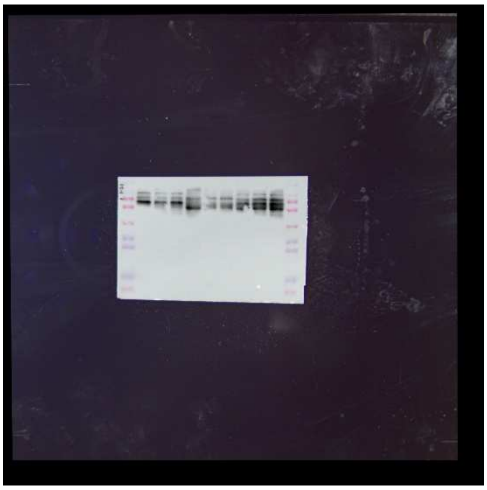
p65

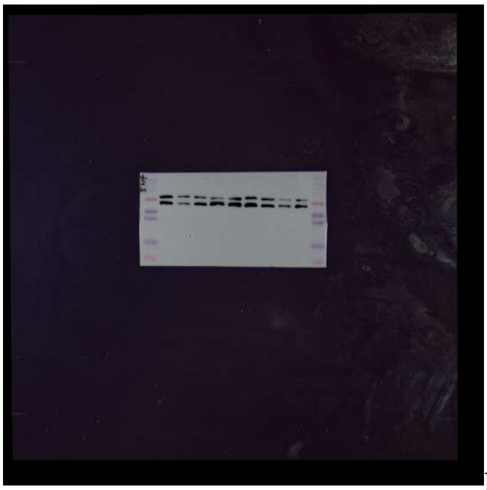
p-IκBα

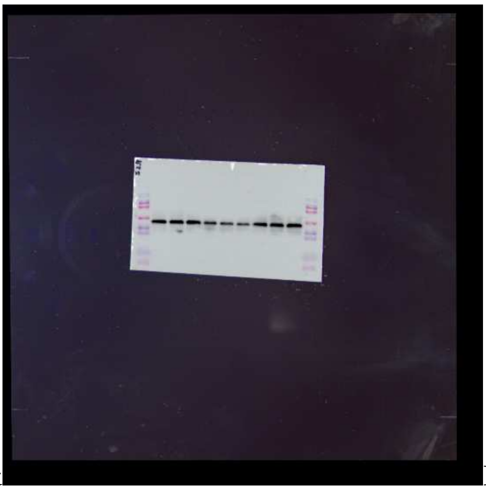
IκBα

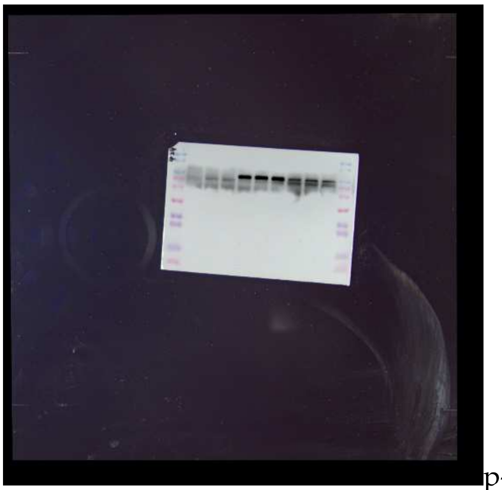
p-IKKα+β

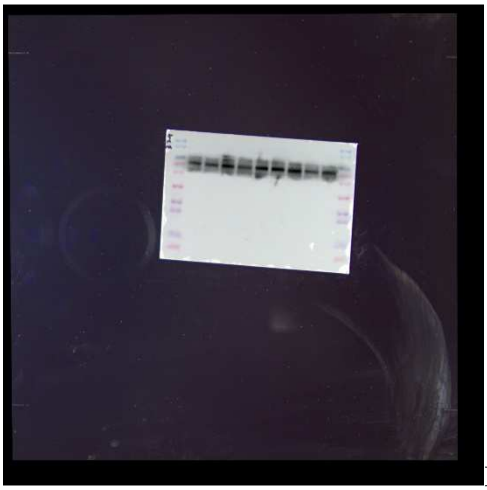
IKKα+β

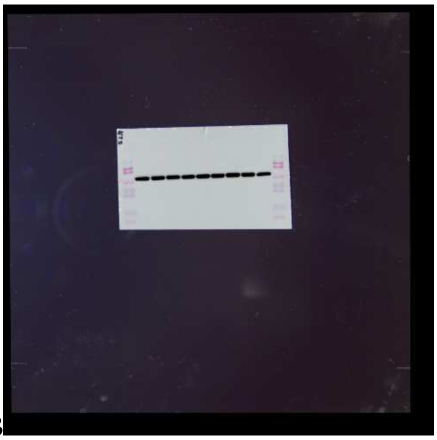
β-actin

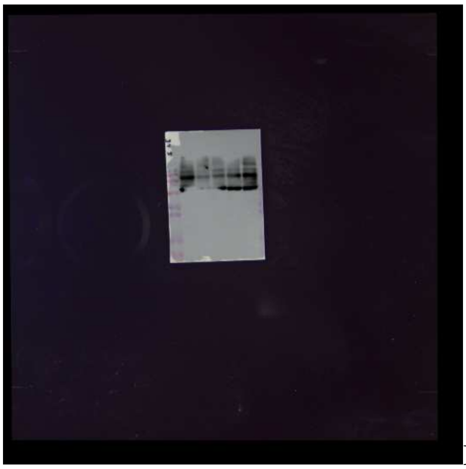
PPARγ

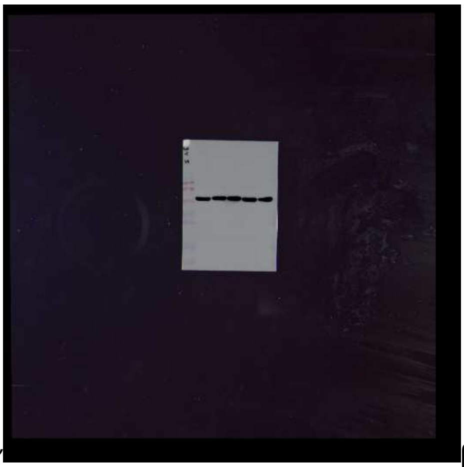
β-actin

2. Western blot data of HC-11 cells. From left to right: Control group, LPS group, Vehicle group, EPA-L group, EPA-M group, EPA-H group.

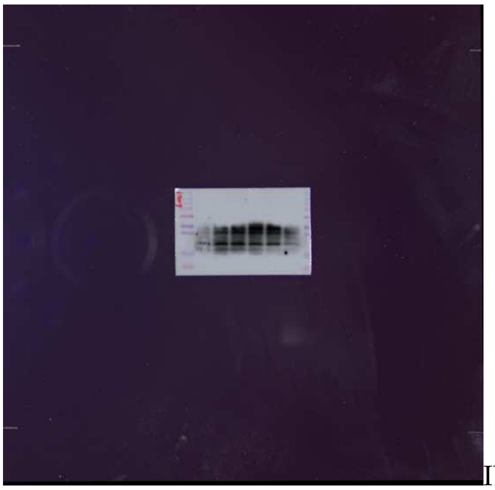
IL-1β

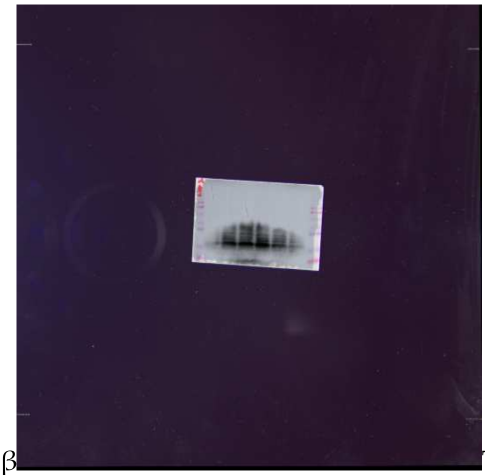
TNF-α

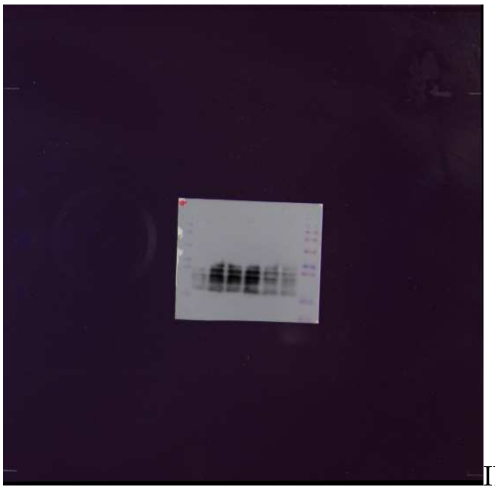
IL-6

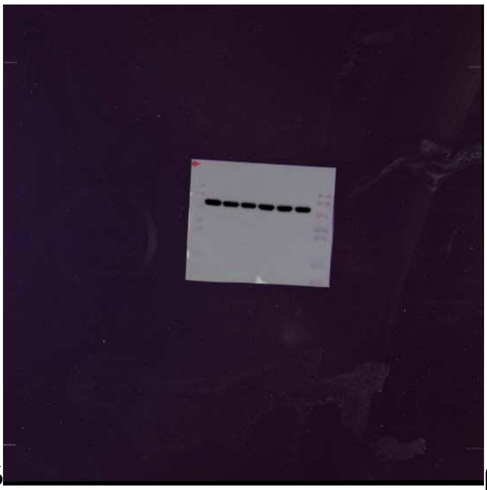
β-actin

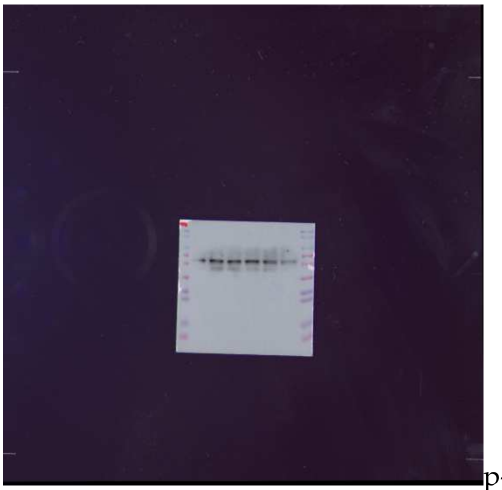
p-p65

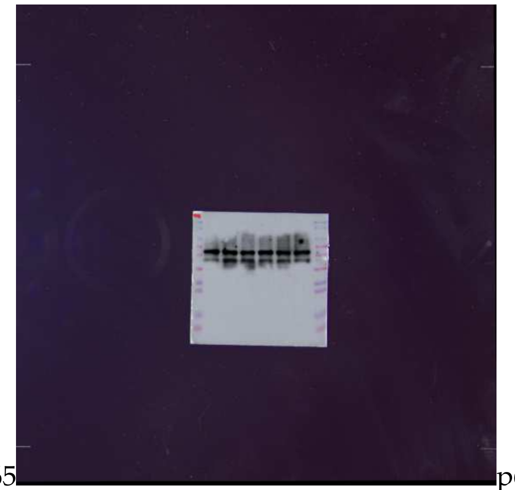
p65

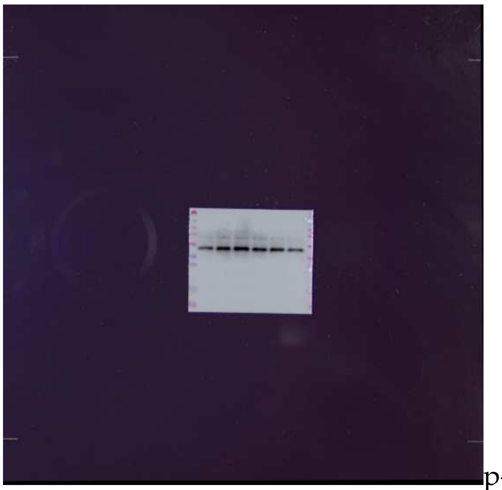
p-IκBα

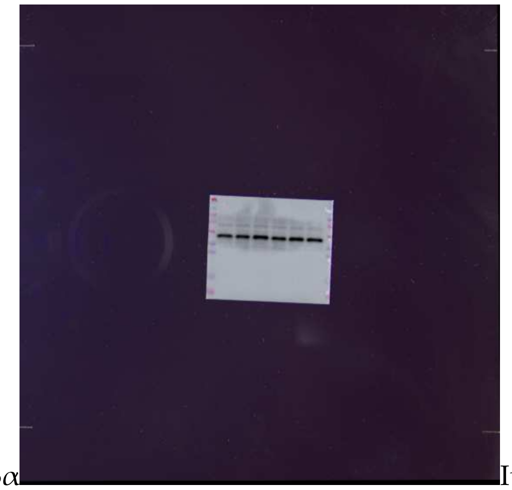
IκBα

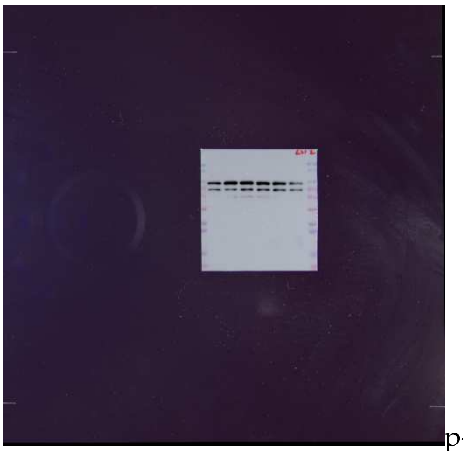
p-IKK α+β

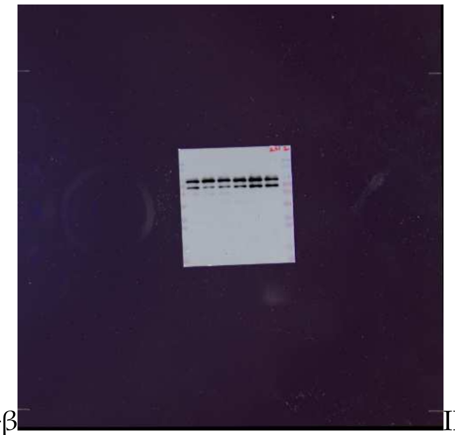
IKK α+β

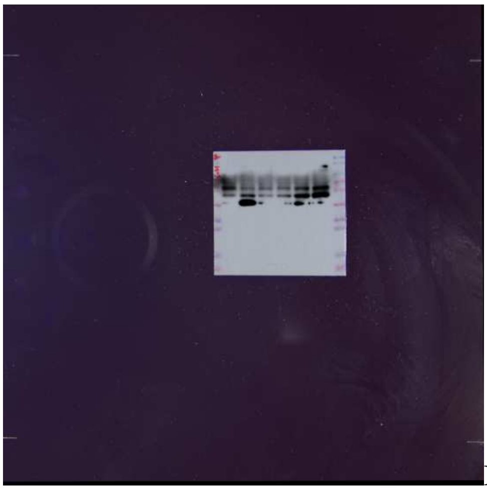
PPARγ

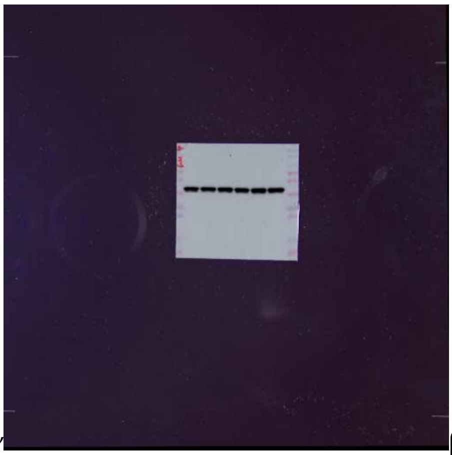
β-actin

3. Western blot data for the PPARγ agonist treatment groups, from left to right: control group, LPS group, EPA+LPS group, pioglitazone + LPS group.

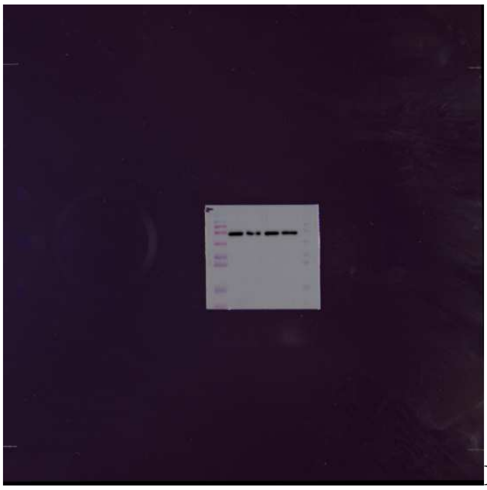
PPARγ

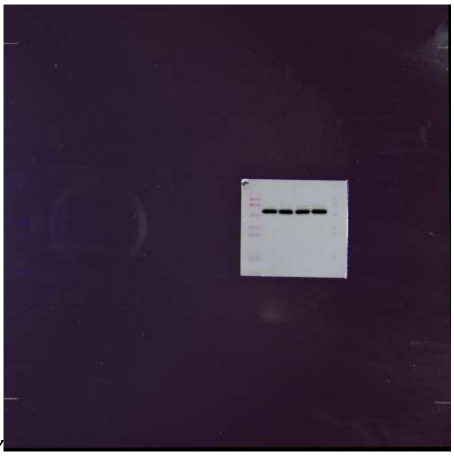
β-actin

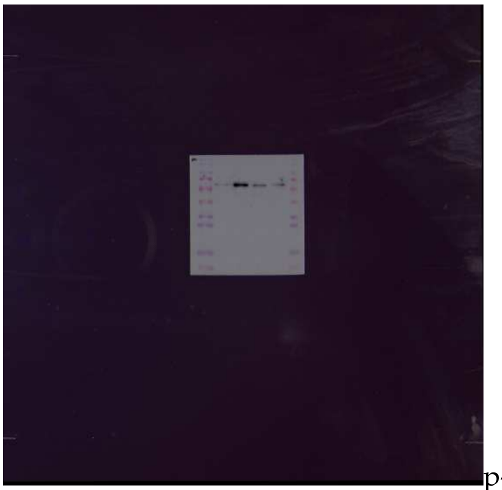
p-p65

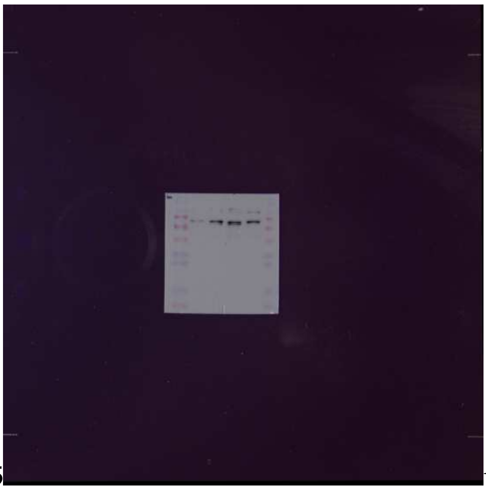
p65

4. Western blot data for the PPARγ inhibitor treatment groups, from left to right: control group, LPS group, EPA+LPS group, GW9662+EPA+LPS group.

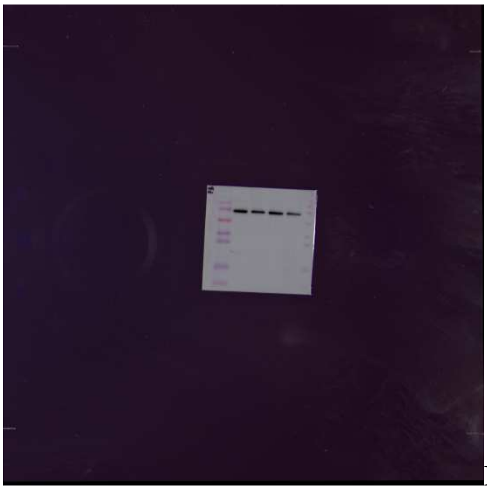
PPARγ

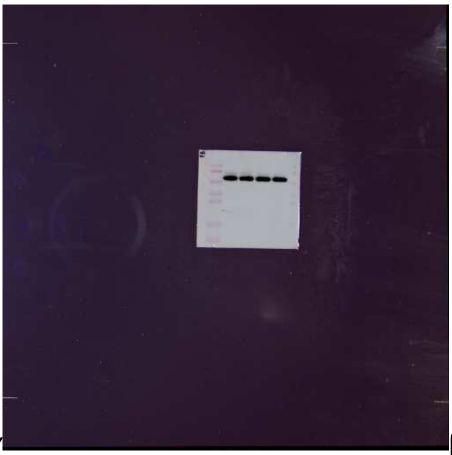
β-actin

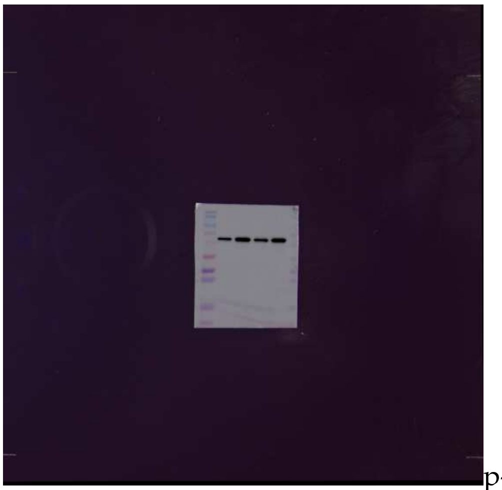
p-p65

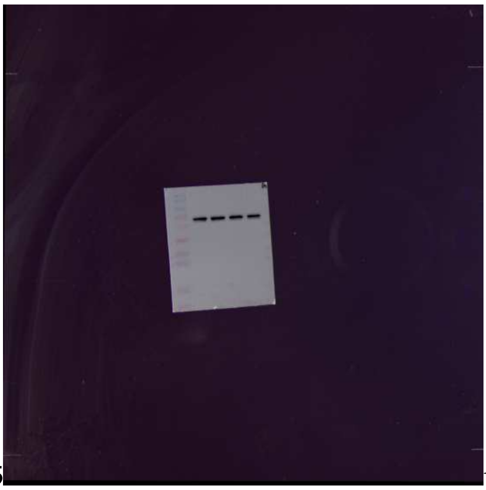
p65
